# Correlation of fasting blood sugar at the time of penile prosthesis surgery with the level of glycated hemoglobin and the outcome of surgery

**DOI:** 10.1186/s12301-021-00198-y

**Published:** 2021-07-02

**Authors:** Mohamad Haobus, Raed Almannie, Mohammed Aziz, Mohammed Farag, David Ralph, John Mulhall, Saleh Binsaleh

**Affiliations:** 1 Urology, Al-Themal Medical Center, Abha, Saudi Arabia.; 2 Division of Urology, Department of Surgery, Faculty of Medicine, King Saud University, Riyadh, Saudi Arabia.; 3 Faculty of Medicine, Urology, Menoufia University, Menoufia, Egypt.; 4 Urology Department, Faculty of Medicine, Al-Azhar University, Assiut, Egypt.; 5 St Peter’s Andrology Centre and the Institute of Urology, UCLH, London, UK.; 6 Sexual and Reproductive Medicine Program, Memorial Sloan Kettering Cancer, New York, NY, USA.

## Abstract

**Background:**

The role of glycemic control in predicting implant infection and other surgical complications is debatable. This study aimed to assess the potential correlation between fasting blood sugar (FBS) levels prior to penile prosthesis surgery (PPS) and the surgical outcomes.

**Methods:**

A retrospective study from data collected prospectively in 2015 in a single center. Patients who underwent penile implant procedures were included. Exclusion criteria were if surgery done by low-volume implanter, patients who required revision surgery or not diagnosed with diabetes mellitus. Management was standardized to all patients.

**Results:**

All complications whether minor or major were documented up to three years. One year after the surgery a Likert scale questionnaire was completed by the patients. In total, 218 patients completed the study at last follow-up. Complications rate was 6.25%. The rate of infection requiring explantation was 3.8%. 0.9% of patients had a superficial infection managed successfully with conservative management. 0.9% had erosion and 0.9% had mechanical failure. There was no statistically significant difference in FBS or glycated hemoglobin (HbA1c) levels in patients with postoperative complications compared to patients with satisfactory postoperative course. FBS level on the day of surgery was within 20 mg/dL (1.11 mmol/L) of the expected range based on HbA1c measurement in 62 patients (28.44%), while in 146 patients (66.98%) the FBS was not within 20 mg/dl (1.11 mmol/L) of the expected range based on preoperative HbA1c level.

**Conclusion:**

FBS levels on the day of surgery are not correlated with HbA1c levels and PPS outcomes.

## 1 Background

Diabetes mellitus (DM) is reaching a pandemic level worldwide, with increasing incidence of young onset type 2 diabetes [1, 2]. The association between DM and the development of erectile dysfunction (ED) has been described more than 200 years ago [3], and the prevalence of ED in diabetic men is ≥ 50%.

The pathophysiology of diabetes-induced ED is multifactorial, and the proposed mechanisms include: elevated advanced glycation end-products, increased levels of oxygen free radicals, impaired nitric oxide synthesis, increased endothelin B receptor binding sites and up-regulated Ras homolog family member A (RhoA/Rho-kinase pathway), neuropathic damage and impaired cyclic guanosine monophosphate (cGMP)-dependent protein kinase-1 [4].

Diabetics men often present with severe ED that is more difficult to treat medically compared with non-diabetic men [1]. The likelihood of undergoing penile implant is doubled in diabetic men having ED compared to non-diabetics [5]. In the Prospective Registry of Outcomes with Penile Prosthesis for Erectile Restoration (PROPPER) study, the authors found that > 20% of men undergoing penile prosthesis surgery were diabetics [6].

Infection remains one of the most devastating complications in penile prosthesis surgery. Large variations in the reported infection rates exist, but in most studies the rate is between 1 and 4%. The incidence of infection reaches 13% for re-implant procedures and up to 21.7% if penile reconstruction was done [6-8].

The role of glycemic control in predicting implant infection is debatable. Some authors believe that diabetes itself is a risk factor for penile implant infection and the risk increases with uncontrolled blood sugar [9-13]. Other authors oppose any association between DM and rate of infections, and they do not recommend glycemic control as a predictor for penile implant infection [14-16]. In a large multicenter prospective study of more than 900 implants by our group, it was found that uncontrolled DM is associated with an increased risk of infection after penile prosthesis surgery. Others also showed that the risk is directly related to glycated hemoglobin (HbA1c) level [16, 17].

DM is a heterogeneous disease in its severity: While some patients are well controlled and do not have significant end-organ damage, others have severe manifestations, e.g., ED, cardiovascular disease, nephropathy, retinopathy and neuropathy. HbA1c is the test that most closely measures how well diabetes is controlled [16]. Many papers were published investigating the correlation between DM and risk of penile implant infection but most of them compared diabetics with non-diabetics [6, 9, 11, 12, 15]. Few available data compared controlled diabetics with non-controlled using HbA1c [8, 13, 16] and only one recent paper investigated blood glucose level before surgery [18].

Our aim in this study is to investigate the relation between fasting blood sugar (FBS) at the time of penile implant and the surgical outcome (complications and patient satisfaction).

## 2 Methods

### 2.1 Study design

This study is based on a prospectively built large database.

### 2.2 Study sitting and study participants

After approval of our local institutional ethics committee, we analyzed the data of penile implant procedures in one full year (2015) in one high-volume facility specialized in andrological surgery. The data collected for each procedure include: surgeon, patient demographics, indication for surgery, procedure-related data (preoperative, intraoperative and postoperative notes) and complications. Peyronie’s disease (PD) was diagnosed primarily clinically by identifying penile plaque and/or deformity during examination. Then this was confirmed with Duplex ultrasonography.

### 2.3 Inclusion criteria

Diabetic patients who underwent penile implant surgery in 2015 were included in this study.

### 2.4 Exclusion criteria

Non-diabetic men, revision surgery, if extra maneuvers are needed (e.g., grafting), if surgery was done by a low-volume penile implantation surgeon. For the purpose of the study, a low-volume surgeon was defined as a surgeon who had performed < 12 penile implant procedures in the study period.

### 2.5 Data collection

The preoperative counseling focused on the goal of surgery, the choice of the device and a detailed explanation of the possible complications. All the complications explained were listed in the consent, which is signed by all patients. The preoperative preparation was standardized and includes: bathing nightly with antibacterial soap for three nights before surgery, avoid shaving which is done in the operating room, administration of 240 mg intravenous gentamycin 2 h preoperatively and 1 g ceftriaxone was given before induction of anesthesia. HbA1c usually done one week before surgery. A blood sample to check fasting blood sugar was taken when patient admitted in the early morning the day of surgery.

All procedures in this study were done under spinal anesthesia. Shaving was done by razor and the prepping was with chlorhexidine–alcohol. For inflatable implants (IPP), all procedures were done through penoscrotal approach. For malleable implants (MPP), the preferred approach was a ventral raphe incision. Postoperatively, patients were admitted for one day. After discharge the follow-up protocol was: twice a week for the first 2 weeks, weekly for weeks 3 and 4, and every 3 months for one year. Infection diagnosis was based on clinical judgment, and all infections were confirmed at the time of explant surgery using bacterial and fungal cultures.

Patient satisfaction was measured by a Likert scale question, which was done at 1-year follow-up. The patients were asked about the overall satisfaction. The answer was scored from 1 to 5 where 5 was very satisfied, 4 satisfied, 3 neutral, 2 dissatisfied and 1 very dissatisfied.

### 2.6 Data analysis

Statistical analyses were conducted using STATA version 13 (StataCorp. 2013. Stata Statistical Software: Release 13. College Station, TX: StataCorp LP.). The t-test, Mann–Whitney U test, Pearson’s Chi-square and Fisher’s exact test were used as appropriate. Multivariate logistic regression was used to assess for independent predictors of infection.

### 3 Results

In total, 218 patients were included in this study. Eighty-five patients underwent IPP and 133 patients underwent MPP. For all MPP, the device was Genesis (Coloplast Corporation, Minneapolis, MN, USA). For IPP, 77 devices were Titan (Coloplast Corporation, Minneapolis, MN, USA), 4 were LGX and 4 were Ambicor (American Medical Systems, Minnetonka, MN, USA). The patient characteristics are summarized in Table 1.

The infection rate was 4.6% (10 infected implants that were all removed). Eight were MPP (6% infection rate), and two were IPP (2.4% infection rate). Both infected IPP were Coloplast Titan. Two patients had superficial wound infection (0.9%) with successful conservative management (one IPP and one MPP). Two patients with Coloplast Titan OTR had pump failure (2% of IPP cases) needed reoperation. Two patients had erosion, and one of them had associated infection. (Both were MPP.) Two patients had mechanical failure, and one of them was associated with infected hematoma. (Both were IPP.) There was no statistically significant difference in FBS in patients with unremarkable postoperative course (152 mg/dL–8.4 mmol/L) compared to those who had major complications (183 mg/dL–10.17 mmol/L, *P* = 0.07). Table 2 shows the list of major complications and their rate based on glycemic control, BMI, device type and the presence of Peyronie’s disease.

The majority of patients were very satisfied or satisfied (88%) (Table 3). Fourteen patients (6.5%) were neutral; they were patients who had complications that were treated conservatively. The remaining patients (5.5%) were dissatisfied or very dissatisfied; those were patients who had a major complication requiring removal of the implant.

## 4 Discussion

Penile prosthesis implantation is the gold standard treatment for patient’s refractory to other less invasive therapy, such as phosphodiesterase type 5 (PDE-5). The rates of infection have decreased over the past decade, because of improvements in device technology like antibiotics- and hydrophilic-coated implants. But infection is still the most feared complication. It often demands reoperation and can increase the cost of care as much as sixfold [19].

Diabetics are at high risk for infections, due to immune dysfunction, diabetic neuropathy and poor circulation. However, it remains unclear whether diabetes significantly increase rate of infection after penile prosthesis implantation. Lipsky et al studied DM as a potential risk factor for IPP infection, and the study included 14,969 patients who underwent initial IPP implantation from 1995 to 2014. About 30% of the cohort were diabetic. The overall infection rate was 343/14,969 (2.3%). Infectious complications were experienced by 3% (133/4,478) of diabetic patients and 2% (210/10,491) of non-diabetic patients (*P* < 0.001). They concluded that DM is a risk factor for IPP infection. They also raised the question of whether this increased risk can be mitigated by optimization of glycemic control before surgery [20].

On the other hand, Osman et al. retrospectively analyzed 875 diabetic patients who underwent primary penile prosthesis implantation from 18 high-volume penile prosthesis implantation surgeons throughout the USA, Germany, Belgium and South Korea. Preoperative HbA1c and blood glucose levels within 6 h of surgery were assessed in univariate and multivariate models for correlation with postoperative infection, revision and explanation rates. They did not find any correlation between preoperative blood glucose levels or HbA1c levels and postoperative infection rates: *p* = 0.220 and *p* = 0.598, respectively. The same group on multivariate analysis found that a history of diabetes-related complication was a significant predictor of higher revision rates (*p* = 0.034), but was nonsignificant for infection or explantation rates [18].

In the present study, we analyzed the data of penile implant procedures in one full year (2015) in a high-volume center specialized in penile prosthesis surgery. Exclusion criteria included non-diabetics, revision surgery and procedures done by low-volume surgeons to minimize other predictors of postoperative complications. Preoperative, intraoperative and postoperative protocols (infection control protocols) were identical for all patients.

All minor (local edema, ecchymosis, pain) and major (infection, erosion, mechanical failure) complications as early as the 1st postoperative week and followed up to 3 years were recorded.

Our results showed that FBS level (on the day of surgery) does not correlate with the rate of complications when compared with patients with unremarkable postoperative course (see Table 2). Also, there was no correlation between FBS level with patient satisfaction (see Table 3).

Strengths of the present study include its relative unique nature because it was performed in a geographic location notorious for a high rate of DM in addition to other comorbidities like obesity and hypertension. All the procedures were standardized in terms of preoperative, operative and postoperative protocols in a high-volume center specialized for penile implants.

However, this study has some limitations: firstly the relatively low number of infected implants and the somewhat limited nature of the Likert scale used to assess patient satisfaction.

## 5 Conclusions

No correlation between day of surgery FBS and outcome (complications and patient satisfaction) of penile prosthesis surgery, or with HbA1c of same cohort of patients.

## Figures and Tables

**Table 1:** Patient characteristics

Data	Result
Mean age (years)	57.7
Mean BMI (kg/m2)	29.8
Mean HbA1c (%)	7.9
Expected FBS based on HbA1c (mg/dL)	181
Mean day of surgery FBS (mg/dL)	154
Patients with Peyronie’s disease	35%
Current smoker	21%
Exsmoker	7.6%
HTN	38%
Ischemic heart disease	10.5%
Malleable prosthesis	61%
Inflatable prosthesis	39%

The data are expressed as mean, number and percent (%), according to the statistic used. *BMI* body mass index, *HbA1c* glycated hemoglobin, *FBS* fasting blood sugar, *HTN* hypertension

**Table 2:** Rate of major complications based on glycemic control, BMI, device type and the presence of Peyronie’s disease

	No complications (*N* = 195)	All infections (*N* = 12)	Infection requiring removal (*N* = 10)	Erosion (*N* = 2)	Pump failure (*N* = 2)
HbA1c (mean) (%)	7.9	8.6 *(*p* = 0.06)	7.7	7.9	7.3
FBS (mean) (mg/dL)	152	183 *(*p* = 0.07)	176	200	133
BMI (mean)	29.8	30.9 *(*p* = 0.73)	29.5	26.1	31
Inflatable prosthesis	94%	4.5%	1.5%	0%	2%
Malleable prosthesis	92%	4.9%	4.9%	1.4%	–
Peyronie’s	36%	18%	25%	0%	0%

* versus patients without complications

*BMI* body mass index, *HbA1c* glycated hemoglobin, *FBS* fasting blood sugar

**Table 3:** Satisfaction scale

Scale	Number	Mean HbA1c	FBS (mg/dL)
5	137	7.9	152
4	55	7.9	155
3	14	8	168
2	4	7.7	151
1	8	7.1	144

*HbA1c* glycated hemoglobin, *FBS* fasting blood sugar

## Abbreviations

FBS: Fasting blood sugar
PPS: Penile prosthesis surgery
HbA1c: Glycated hemoglobin
DM: Diabetes mellitus
ED: Erectile dysfunction
cGMP: Cyclic guanosine monophosphate
IPP: Inflatable penile implants
MPP: Malleable penile implants
PDE-5: Phosphodiesterase type 5
